# The genetics of infectious disease susceptibility: has the evidence for epistasis been overestimated?

**DOI:** 10.1186/1741-7007-11-79

**Published:** 2013-07-15

**Authors:** Matthew D Hall, Dieter Ebert

**Affiliations:** 1 University of Basel, Zoological Institute, Vesalgasse 1, Basel, CH-4051, Switzerland

## Abstract

Interactions amongst genes, known as epistasis, are assumed to make a substantial contribution to the genetic variation in infectious disease susceptibility, but this claim is controversial. Here, we focus on the debate surrounding the evolutionary importance of interactions between resistance loci and argue that its role in explaining overall variance in disease outcomes may have been overestimated.

## Opinion

Differences amongst individuals in their susceptibility to infection seldom have a simple genetic basis and are often determined by a complex interplay of multiple loci. Characterizing the number, location and effect size of the quantitative trait loci (QTL) underlying this variation informs our understanding of not only the pathways that influence susceptibility, but also the potential coevolutionary dynamics of host and parasites. Of particular interest is the role that epistasis, defined broadly as interactions among loci in determining a phenotype, has in shaping the variation we see in infectious disease susceptibility. For most complex traits, quantitative genetic theory suggests that epistasis is unlikely to contribute substantially to genetic variation [1,2]. However, models of host-parasite co-evolution typically feature some degree of epistasis between resistance loci [3,4], and the results of empirical linkage and association mapping studies suggest that epistatic interactions can explain considerable variation in infectious disease characteristics within natural populations [5,6].

In this article, we will discuss the current state of genetic studies of disease susceptibility, with a particular focus on the theoretical and empirical support for epistasis. We then ask if the genetic basis of infectious disease susceptibility is different from other traits, or if the evidence for epistatic interactions has been overestimated. To provide the necessary background, we first give a brief overview of the debate surrounding the contribution of epistasis to the genetic architecture of complex traits.

## The genetic architecture of complex traits

**Figure 1:**
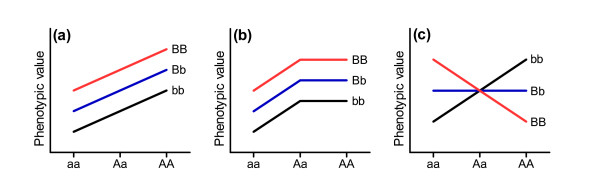
**Models of gene action for complex traits.** Phenotypic values are shown for two diploid host loci under different patterns of gene action. **(a)** Additive effects at locus A and B. Each allele contributes a fixed metric value to the trait, independent of the effects of other alleles at the same or different loci. Human height is a classic example of a complex trait where approximately 80% of the variation in height amongst individuals is due to additive genetic effects [8]. **(b)** Dominance at locus A. Both loci are independent, but with dominance occurring at locus A, as the phenotypic value of the heterozygote is not midway between the values of the two homozygotes. Complete dominance, as shown here, is typical of Mendelian genetic disorders such as Huntington's disease where an affected individual need only inherit one copy of the mutant allele. **(c)** Epistatic interactions between locus A and B. Epistasis is estimated as the deviance from the additive combination of two loci and can take many forms, depending on whether an allele combination is more or less fit than expected. Well known examples of epistasis include the interaction between genes in shaping coat color in mice, or the occurrence of synthetic lethality seen when mutations occur in two genes with redundant functions [13].

A quantitative genetic understanding of complex traits is based on partitioning the variation between individuals that is due to their genotypes into additive, dominance and epistatic components [7]. Each component relates to a different form of gene action, with the additive component describing the variance associated with the independent contribution of alleles, dominance describing the variance contributed by interactions between alleles at the same locus, and epistasis referring to the contribution of interactions between alleles at different loci (Figure 1). While the relative contribution of each of these components to genetic variance depends on the underlying allele frequencies within a population, quantitative genetic theory suggests that most of the genetic variation in a population will be due to the additive effect of allelic substitutions [1]. Yet this assertion is not without controversy. Although the additive genetic basis of a trait can be readily estimated using information on the relatedness of individuals (via known breeding designs or pedigrees), only a fraction of this genetic variation has been linked to underlying loci in genome-wide association studies (GWAS; [8], but see [9]). This ‘missing heritability’, as it has been termed [10], together with the increasing knowledge of modifier genes and gene networks, has led to the suggestion that epistasis may make a substantial contribution to the overall levels of genetic variation [5] and that the contribution of additive variance may have even been overestimated [11].

At the heart of this debate is the assumption that the importance of epistasis at the mechanistic level is reflected in the patterns of phenotypic and genetic variation at the level of the population. Within an individual, interactions between genes can result from a wide range of molecular mechanisms and can have positive or negative effects on fitness, depending on whether the resulting phenotype is greater or less than the individual effects of the alleles [12,13]. This functional impact of gene-gene interactions, however, is different from the statistical contribution of epistasis to complex trait variation within a population [13], because the latter depends on the distribution of allele frequencies in that population [7]. If most alleles are at extreme frequencies, then the majority of genetic variation should still be additive, even if there is dominance or epistasis acting at individual loci [1]. In this case, the effect of rare alleles that interact will be negligible as the likelihood that two rare alleles are present in the same individual is very low. Thus, from a quantitative genetic perspective, it seems unlikely that epistatic interactions will contribute substantially to phenotypic variance unless the alleles are of major effect, and the frequencies of alleles involved in epistatic interactions are intermediate.

## Are complex traits resulting from host-parasite interactions different?

**Figure 2:**
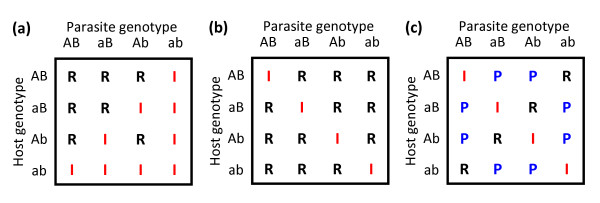
**Genetic models of host-parasite interactions.** Infection outcomes are shown for three different interaction models (haploid hosts and parasites with two loci and two alleles each), where R represents resistant individuals, I represents non-resistant individuals, and P represents partially resistant individuals. **(a)** Gene-for-gene-model. The mechanistic basis of the gene-for-gene model is that resistance by the host requires recognition of a gene product ‘elicitor’ produced by the parasite [15,16]. Thus, a host is resistant if it contains one resistance allele that matches an avirulence allele (A or B) of the parasite. **(b)** Matching-allele model. In an architecture inspired by the self-nonself recognition systems of the animal immune system [14,15], the matching-allele model assumes that a host can resist a parasite unless the parasite matches all of its interacting alleles. **(c)** Multiplicative matching-alleles model. This model is presented as a counterpoint to the matching-allele model, as it does not implicitly involve epistatic interactions between resistance alleles at different loci [4,29]. Instead, the number of matched alleles determines host and parasite fitness in a multiplicative fashion, leading to intermediate, or partial, estimates of resistance.

How then does the genetic architecture of disease resistance compare to other complex traits? Based on models of host-pathogen coevolution, resistance is commonly predicted to involve multiple host genes and strong interactions between alleles at different host loci (Figure 2). Epistasis in the matching-allele class of models, for example, arises because each multi-locus parasite genotype can only infect a corresponding multi-locus host genotype [14,15]. As natural selection favors parasites that match the most common host genotype, overrepresented host genotypes become disproportionately unfit, while rarer allele combinations now have a fitness advantage. This type of selection, whereby the fitness of a genotype decreases as its frequency increases, is known as negative frequency-dependent selection. Conversely, in the gene-for-gene model of host-pathogen interactions [16], epistasis can be incorporated via the costs associated with maintaining multiple resistance alleles (for example, [17]). Here, the costs of resistance either accelerate or decelerate with the number of contributing loci [18], aiding the maintenance of polymorphisms in resistance genes. In both cases, epistasis facilitates the rapid co-evolutionary cycles that are a hallmark of host-pathogen theory [19,20], as recombination can now break-up unfavorable allele combinations, allowing oscillations between host and parasite genotypes under negative frequency-dependent selection.

In support of such genetic models, studies of infectious disease susceptibility have commonly documented three key indicators of epistasis. Both linkage and association mapping studies have shown that variation in measures of resistance are often associated with multiple QTL of major effect, that interactions between loci contribute substantially to phenotypic variation, and that evidence for specific candidate loci is often difficult to replicate in other experiments or environments [6,21]. In a meta-analysis of over 500 QTL mapping experiments, for example, Wilfert and Schmid-Hempel [6] found that epistatic interactions were identified in 48 of 62 studies involving genome-wide scans, with most epistatic loci not previously identified using single QTL analyses (123 of 170 loci). Conventional quantitative genetic studies have also characterized the contribution of non-additive genetic components to patterns of susceptibility and resistance. Using reciprocal crosses between four populations, for example, a study of resistance in the red flour beetle (*Triboliumcastanaeum*) found that epistasis explained significant variation in host survival only upon infection by a parasite, and not under the unexposed and uninfected control conditions [22].

## Has the evidence for epistasis been overestimated?

At first glance, the high prevalence of epistasis in mapping studies of disease traits suggests that interactions amongst host resistance genes might indeed contribute substantially to variation in disease susceptibility, as is assumed by models of host-parasite coevolution. What needs to be taken into account, however, is the estimation bias inherent in conventional mapping studies. Minor effect variants, for example, are unlikely to be identified in linkage studies due to a combination of broadly spaced markers and limited sample sizes. Conversely in association mapping, rare alleles will be difficult to detect due to the reliance on linkage disequilibrium between common markers and common causative variants (see discussions in [23]). Gene frequencies are also altered as part of the design of traditional mapping panels, which typically involve some level of inbreeding, combined with crosses between a few individuals representing phenotypic or even population extremes. In an F2 inter-cross between high and low resistance genotypes, for example, allele frequencies are on average 0.5, even if variants within the mapping panel were at extreme frequencies in the original population. Thus, by concentrating or combining alleles of major effect within and between populations, conventional mapping studies bias allele frequencies towards intermediate values and therefore increase the chance of finding epistasis.

Without information on the effect size and frequency of alleles in natural populations, it is difficult to determine whether epistatic interactions contribute substantially to genetic variation in quantitative susceptibility, or if the contribution of such interactions to individual variation has been overestimated. The lack of success in identifying the same loci across different experiments, for example, could be due to epistasis between resistance loci, or the result of the strong sampling bias and small fraction of genetic variation that is captured using experimental crosses. Nonetheless, identified epistatic interactions can be functionally important. In a number of studies, gene-gene interactions have helped characterize the pathways underlying the mechanisms of resistance (for example, [24-27]). In the mouse, for example, epistatic interactions revealed a new mechanism for resistance to the mouse cytomegalovirus, which involves an interaction between a receptor for natural killer cells and a molecule of the major histocompatibility complex on virus-infected cells [28]. Such studies highlight the functional utility of characterizing epistasis, even if the statistical contribution of each gene-gene interaction to variation in a complex trait remains unclear.

## Reconciling quantitative genetic and host-parasite theory

While epistasis is an integral component of many models of disease resistance and antagonistic coevolution (but not all [4,29]; Figure 2), the contribution of epistatic variance to susceptibility remains difficult to evaluate using conventional QTL mapping methods. With the advent of next generation sequencing approaches, however, new insights can be generated into the genetic architecture of susceptibility [23,30]. GWAS, for example, allow for the total genetic variation within a population to be decomposed into the combined effect of all loci acting additively (for example, [9]). The remaining, unexplained genetic variation, therefore, gives an upper limit for how much epistasis could potentially contribute to variation in infectious disease [1]. Observed allele frequencies and effect-size parameters can also be estimated for a range of susceptibility loci (sensu [31]), and then compared to the expected intermediate allele frequencies predicted by different models of host-parasite coevolution. Yet, higher marker densities and GWAS do not completely resolve the contribution of specific gene-gene interactions to trait variation. Pairwise epistatic interactions are difficult to evaluate using the hundreds or thousands of makers required for conventional QTL mapping studies, let alone using the millions of markers required for GWAS.

Even in model systems where next generation sequencing approaches have been used extensively, we are far from a general understanding of the underlying architecture of resistance and susceptibility. In *Drosophila*, for example, considerable progress has been made in identifying loci underlying resistance to sigma virus transmission and verifying the importance of the resistance alleles in natural populations [32-35]. Yet resistance genes, such as *ref(2)P*, are often strain specific and do not completely account for the genetic variance underlying resistance to multiple virus isolates [36]. Similarly, despite the wide range of infectious diseases that have been studied in humans using high-density genetic maps [37,38], debate is still ongoing as to the distribution of allelic variants, and whether the genetic basis of susceptibility is based on high frequency common variants or the cumulative effects of many rare mutations [30]. As such, we suggest that two key aspects of host-parasite biology will need more consideration as we move forward in the genomics era: first, that our understanding of the genetics of susceptibility will depend on the number of parasite genotypes included in association studies; and second, that the expectation of epistasis may not be appropriate for all measures of resistance.

How we account for the natural genetic diversity of parasites will strongly influence our understanding of the genetic architecture of susceptibility. If the causal parasite is unknown or resistance is assessed using a mix of parasite genotypes, then mechanisms unrelated to resistance could be contributing to variation in infectious disease. Competition between multiple parasite genotypes within the host [39,40] and variation in dose-dependent effects across isolates [41] are all processes that would bias infection estimates. Conversely, if only a single pathogen genotype is used in a mapping study, then the relevance of any candidate loci is difficult to extend beyond the response of the host to that specific genotype. Indeed, where multiple strains of a parasite have been utilized within a mapping or association study, the results suggest that only a subset of identified QTL will confer resistance to all genotypes [6]. A study exploring the association between mosquito immune genes and infection by *Plasmodium falciparum*, for example, revealed that certain candidate loci explained patterns of resistance only for specific parasite isolates [42]. These findings highlight the need to account for the contribution of parasite genetic variation to variation in host susceptibility, otherwise the genetic architecture of disease susceptibility will be misrepresented.

Careful consideration of the trait used to characterize resistance will also be important for mapping studies. Phenotypes of resistance range from infection rates and parasite loads, through to symptoms of disease such as morbidity and mortality. Underlying each of these measures will be a range of processes involving the ability of a pathogen to penetrate the host, the recognition of parasite proteins by the host, and the subsequent immune response facilitating pathogen replication [43,44]. Thus, the type of trait used to estimate resistance and the timing of a phenotypic assay (early or late in the infection process) could significantly influence the characterization of phenotypic and genetic variance. Estimating resistance based on symptoms of disease, for example, may more closely match classical quantitative genetic theory, whereas the initial ability of a parasite to penetrate a cell or tissue is a better fit for models of host resistance where epistasis features strongly. Indeed, initial infectivity in plants is often highly specific to certain host-parasite combinations, suggesting that susceptibility/resistance may be under control of a few major genes [45]. Although such insights are uncommon in animals, studies are beginning to reveal that initial resistance to certain pathogens may follow a similar pattern [46], with subsequent symptoms of disease being more quantitative [47].

In summary, the contribution of epistasis to phenotypic and genetic variation is a complex issue for studies of host-parasite interactions. Unlike other quantitative traits, where theory points to the largely additive contribution to genetic variation [1], epistasis is a key component of many models of host-parasite interactions. As such, host-parasite research has focused on characterizing epistasis between resistance loci, rather than debating and evaluating the relative contributions of additive and epistatic genetic effects to phenotypic variance. Nonetheless, as more studies characterize the allelic variants underlying quantitative susceptibility in natural populations, the opportunity to reassess the importance of epistasis will help redefine how empirical and theoretical research approaches the genetic architecture of host-parasite interactions.
